# Automation of a flow-injection system for multispeciation

**DOI:** 10.1155/S1463924686000159

**Published:** 1986

**Authors:** Juan Ruz, Antonia Torres, Angel Ríos, M. D. Luque de Castro, Miguel Valcárcel

**Affiliations:** Department of Analytical Chemistry Faculty of Sciences University of Córdoba Spain


Journal of Automatic Chemistry/*Journal of Clinical Laboratory Automation*, Vol. 8, No. 2 (April–June 1986), pp. 70–74

### Automation of a flow-injection system for multispeciation

**Juan Ruz, Antonia Torres, Angel Ríos, M. D. Luque de Castro and Miguel Valcárcel**

*Department of Analytical Chemistry, Faculty of Sciences, University of Córdoba, Spain*

### Introduction

Differentiating between the two most frequent oxidation states of chromium is fundamental in speciation studies of this element on account of the toxicity of hexavalent chromium. In fact, most of the works on this element published so far deal solely with this aspect [1]. Flow-injection analysis (FIA) has been used several times for determining this element in one [2 and 3] or in its two most common oxidation states [4–10].

Nevertheless, it is known that there is a large variety of chemical forms of Cr(III) and Cr(VI), which show different properties depending on the groups or elements to which they are bound. Their relative abundance is a function of the type of medium. Since the pH is a major parameter in any type of solution (especially in aqueous ones) and taking into account that its value dictates the predominance of a particular species of Cr(III) and Cr(VI) (hydroxylated, dimer etc.), the following species of chromium must be considered to be present in waters. Chromium(III): $Cr(H_2O)_6^{3+}$, $Cr(OH)^{2+}$, $Cr(OH)_2^+$, $Cr(OH)_3$, $Cr(OH)_4^-$. Chromium(VI): $H_2CrO_4$, $HCrO_4^-$, $Cr_2O_7^{2-}$, $CrO_4^{2-}$. Among these species, $Cr(OH)_3$ is very insoluble, its concentration in water being negligible, and $H_2CrO_4$ is a relatively strong acid ($pK_a = -1{\cdot}0$), which is unlikely to occur in water. Consequently the following equilibria, with their respective constants, must be considered:

*Chromium(VI)*

$$H_2CrO_4 \rightleftharpoons HCrO_4^- \qquad pK_1 = -1{\cdot}0$$
$$HCrO_4^- \rightleftharpoons CrO_4^{2-} + H^+ \qquad pK_2 = 6{\cdot}5$$
$$Cr_2O_7^{2-} + H_2O \rightleftharpoons 2\,HCrO_4^- \qquad pK_3 = 1{\cdot}5$$

*Chromium(III)*

$$Cr^{3+} + H_2O \rightleftharpoons Cr(OH)^{2+} + H^+ \qquad pK_4 = 3{\cdot}95$$

$$Cr(OH)^{2+} + H_2O \rightleftharpoons Cr(OH)_2^+ + H^+ \qquad pK_5 = 5{\cdot}50$$

$$Cr^{3+} + 3\,OH^- \rightleftharpoons Cr(OH)_3 \qquad pK_6 = 30{\cdot}00$$

$$Cr^{3+} + 4\,OH^- \rightleftharpoons Cr(OH)_4^- \qquad pK_7 = -29{\cdot}00$$

The pH range over which each species predominates can be conveniently inferred from plots of log/concentration/ versus pH [11].

The authors have used the FIA technique together with computerized numerical calculation methods to study the distribution of the type of water analysed and the above-mentioned pK values. By adding a microprocessor to the FIA system, and creating a suitable calculation program, the different equations established can be conveniently solved. The occurrence in the medium of complexing species for chromium, and/or the presence of a colloid resulting in adsorption phenomena for chromium ions, have not been taken into account. Including these would require information about of the type of ligand and/or colloid, their concentration and corresponding equilibrium constants; additionally, the calculation program would need to be modified.

The value of the ionic strength of the sample must be taken into account because it affects the values of the constants. Constants with an ionic strength of 0·1 M have been used by the authors. The reversed FIA, rFIA [9], and asymmetric merging zones [9] modes have also been utilized, together with the indicator reaction reported by several authors [3–10] involving Cr(VI) and 1,5-diphenylcarbazide.

There is only one previous report on this type of work: a recent theoretical study on the effect of the pH on the Cr(VI) species present in solution [12]. This was carried out by the conventional technique and Cr(III) species were not considered.

### Experimental

*Reagents:* aqueous stock solution of: Ce(IV) (0.500 g $l^{-1}$), Cr(III) (100 μg $ml^{-1}$), Cr(VI) (100 μg $ml^{-1}$) and $H_2SO_4$ (1 M). 1,5-diphenylcarbazide (1,5-DPC): 0.425 g were dissolved into 100 ml of ethanol and diluted up to 250 ml with distilled water.

*Apparatus:* a Pye-Unicam SP6-500 single-beam spectrophotometer equipped with Hellma 178·12QS flow-cell (inner volume 18 μl) and Radiometer REC 80 recorder; FIAtron 721 flow-cell with glass-calomel microelectrode; Hewlett-Packard HP-85 microprocessor with HP-IB 82937A interface and HP 3478A multimeter; Gilson Minipuls-2 and Ismatec S-840 peristaltic pumps, Rheodyne 5041 and dual home made injection valves with variable injection volumes and Tecator TM III chemifold were used.

*Manifold:* the pH of the water sample, which travels through the system, is continuously monitored (glass-calomel microelectrode –ME– in figure 1), prior to its confluence with a sulphuric acid stream, whereupon the stream is split into two channels with an injection valve each. The oxidant (Ce[IV]) is injected into channel 1, while 1,5-DPC is injected into channel 2. The reactor lengths and injection volumes are optimized in such a manner that the confluence of the injected plugs at point A is asymmetric, the plug travelling along channel 1 and

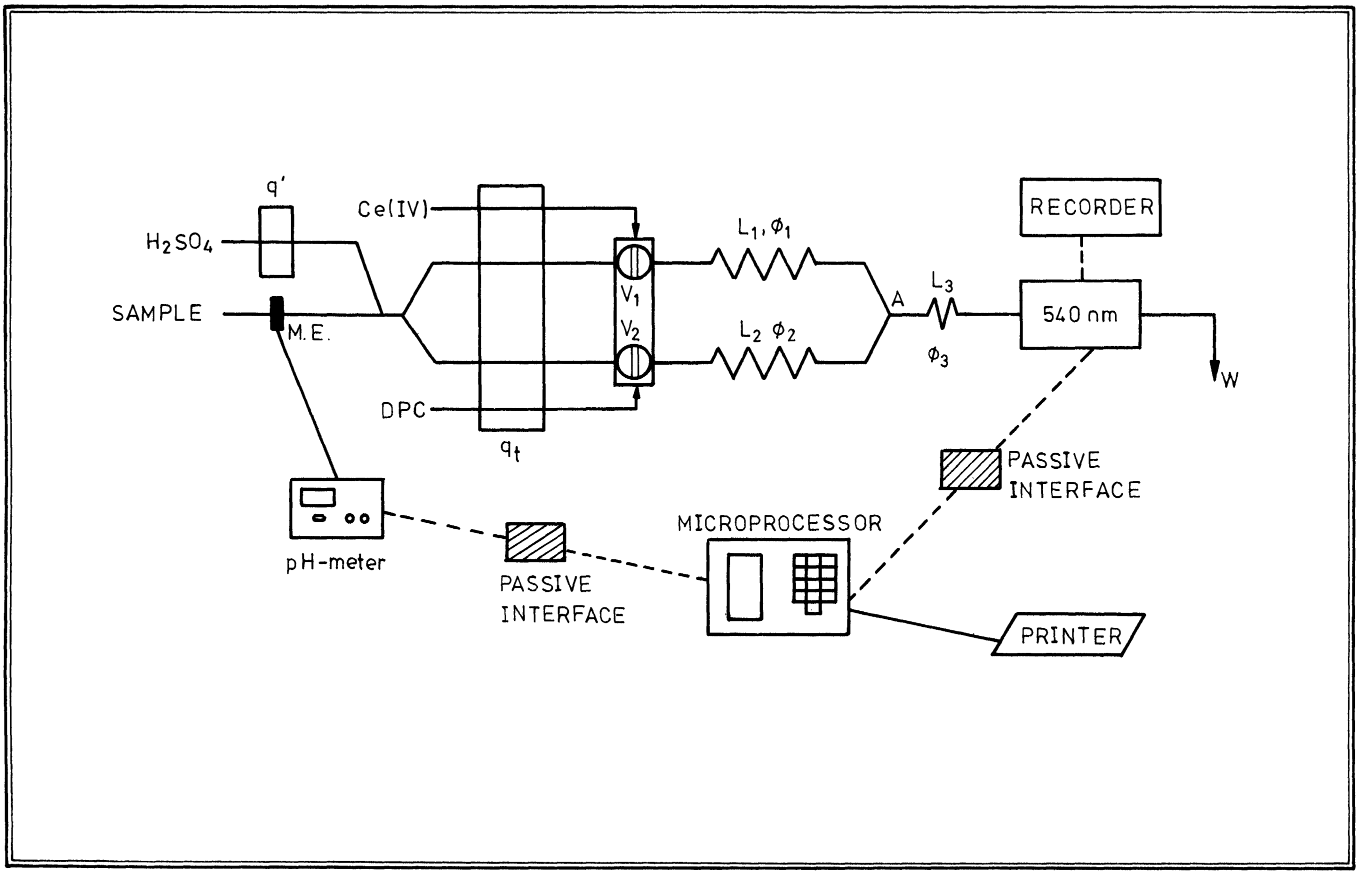

*Figure 1. Diagram of the system used for chromium speciation studies. Optimum working conditions: /DPC/ 0·17% (w/V), /Ce(IV)/ 0·5 g.* $l^{-1}$*, /$H_2SO_4$/ 1 M, $q_t$ 3.28 ml.min$^{-1}$, q' 0·30 ml.min$^{-1}$, $V_1$ 130 µl, $V_2$ 475 µl,* $L_1$ *650 cm,* $L_2$ *360 cm,* $L_3$ *60 cm, $Ø_1$ = $Ø_2$ = $Ø_3$ = 0·5 mm.*

merging with the tail of the plug circulating through channel 2. A plug with two reaction zones is formed in reactor $L_3$. The first of such zones corresponds to the reaction between 1,5-DPC and Cr(VI) from the sample, while the second one is due to the overall chromium content (Cr[III] being previously oxidized to Cr[VI] by injected Ce[IV]). The detection of this plug provides two peaks per simultaneous injection, which are related to the Cr(VI) concentration and the total chromium present in the samples, respectively. The presence of two passive interfaces between the two detectors and the microprocessor means that pH and absorbance data can be acquired and processed. A printer provides the results, which are expressed as the concentration of each species.

The optimization of physico-chemical and FIA variables affecting the system provides the results shown in figure 1's caption. The calibration of the microelectrode with suitable buffers was performed at the working flow-rate, owing to fact that the response of this sensor is affected by the flow velocity.

### Principle behind the calculation

The suggested configuration (figure 1), allows the pH value for each sample to be directly obtained and the Cr(III) and Cr (VI) content from the double peak to be obtained on each simultaneous injection of reagents. Three calibration curves must be previously run:

(1) Curve for Cr(VI) from the absorbance of the first peak, $A_1$, which allows the direct determination of this species in the sample.

(2) Curve for the contribution, $A_2$, of Cr(VI) from the sample to the absorbance of the second peak.

(3) Curve for the contribution, $A_2'$, of Cr(III) from the sample to the absorbance of the second peak.

The equations of these straight lines, regression coefficients and determination ranges found are as follows.

$$A_1 = 0{\cdot}553|Cr^{6+}|+0{\cdot}003 \quad (r = 0{\cdot}9999) \quad 0{\cdot}20 \leqslant |Cr^{6+}| \leqslant 1{\cdot}20 \quad (\mu g\ .\ ml^{-1})$$

$$A_2 = 0{\cdot}450|Cr^{6+}|-0{\cdot}022 \quad (r = 0{\cdot}9959)$$

$$A_2 = 0{\cdot}247|Cr^{3+}|+0{\cdot}018 \quad (r = 0{\cdot}9979) \quad 0{\cdot}50 \leqslant |Cr^{3+}| \leqslant 3{\cdot}00 \quad (\mu g\ .\ ml^{-1})$$

The calculation program discussed below allows, from a series of Cr(III) and Cr(VI) standards, the above-mentioned calibration curves to be established; and, hence, the concentrations of both oxidation states of chromium can be calculated. These data, together with the pH for each sample, permits the determination of the concentration of the different species on the basis of the following equations:

*Chromium(VI)*

$$|HCrO_4^-| = \frac{-B_2 \pm \sqrt{(B_2)^2 + 8\,K_3|Cr^{6+}|overall}}{4\,K_3} \qquad (1)$$

$B_2$ being equal to: $1 + \dfrac{|H^+|}{K_1} + \dfrac{K_2}{|H^+|}$

$$|H_2CrO_4| = \frac{|HCrO_4^-|\ |H^+|}{K_1} \qquad (2)$$

$$|CrO_4^{2-}| = \frac{K_2|HCrO_4^-|}{|H^+|} \qquad (3)$$

$$|Cr_2O_7^{2-}| = 2\ K_3|HCrO_4^-|^2 \qquad (4)$$

*Chromium(III)*

$$pH = 14 + \frac{1}{3}(\log P_s - \log|Cr^{3+}|) \qquad (5)$$

$$S = \frac{P_s}{|OH^-|^3} + K'\,|OH^-| \qquad (6)$$

$$K' = K_7 P_s \qquad (7)$$

$$|Cr(OH)_4^-| = K'\,|OH^-| \qquad (8)$$

The fraction, $F$, of precipitated Cr(III) being:

$$F = |Cr^{3+}| - S \qquad (9)$$

$$|Cr(OH)^{2+}| = M/B_1 \qquad (10)$$

$B_1$ and $M$ being: $B_1 = 1 + \dfrac{|H^+|}{K_4} + \dfrac{K_5}{|H^+|}$ ; $M = S - |Cr(OH)_4^-|$ (where $S$ = solubility).

$$|Cr^{3+}| = \frac{M}{B_1}\ \frac{|H^+|}{K_4} \qquad (11)$$

$$|Cr(OH)^{2+}| = \frac{M}{B_1}\ \frac{K_5}{|H^+|} \qquad (12)$$

Equations (1) to (12) are used in the calculation program, MECROM (see flow diagram in figure 2). The results are given below; for the definition of the variables see the Appendix.

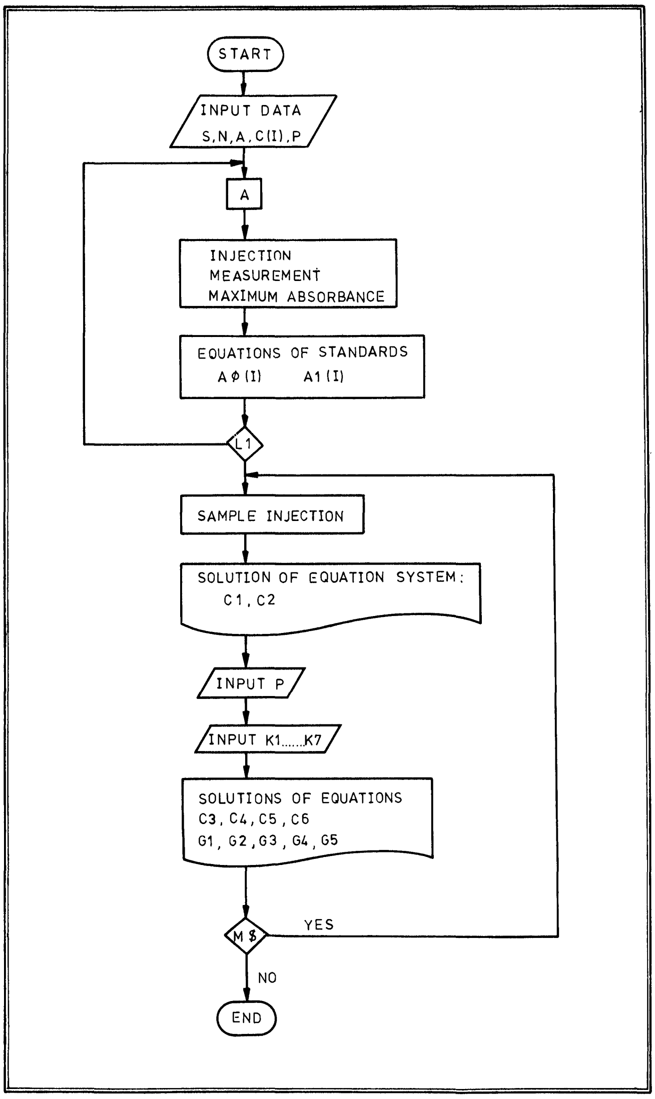

*Figure 2. Flow diagram of the MECROM program for speciation studies of chromium (for definition of variables see appendix).*

### Results

Table 1 shows the values corresponding to several synthetic water samples containing different chromium

*Table 1. Chromium speciation with the suggested configuration and using the MECROM program.*

| Added (μg ml$^{-1}$) | | | Found (μg ml$^{-1}$) Species of Cr(III) | | | | | | Species of Cr(VI) | | |
|---|---|---|---|---|---|---|---|---|---|---|---|
| Cr(III) | Cr(VI) | pH | $Cr(H_2O)_6^{3+}$ | $Cr(OH)^{2+}$ | $Cr(OH)_2^+$ | F* | $Cr(OH)_4^-$ | $H_2CrO_4$ | $HCrO_4^-$ | $Cr_2O_7^{2-}$ | $CrO_4^{2-}$ |
| 3·000 | 0·200 | 4·51 | 0·573 | 2·032 | 0·203 | — | — | — | 0·200 | — | — |
| 2·500 | 0·400 | 4·58 | 0·497 | 1·763 | 0·176 | — | — | — | 0·411 | — | 0·004 |
| 2·000 | 0·600 | 4·65 | 0·287 | 1·440 | 0·203 | — | — | — | 0·600 | — | 0·008 |
| 1·500 | 0·800 | 4·78 | 0·158 | 1·072 | 0·204 | — | — | — | 0·792 | 0·002 | 0·015 |
| 1·000 | 1·000 | 4·95 | 0·069 | 0·696 | 0·196 | — | — | — | 0·960 | 0·002 | 0·027 |
| 0·500 | 1·200 | 5·24 | 0·015 | 0·290 | 0·160 | — | — | — | 1·138 | 0·003 | 0·064 |
| 0·500 | 0·500 | 5·26 | 0·015 | 0·309 | 0·178 | — | — | — | 0·486 | 0·001 | 0·028 |
| 2·000 | 1·200 | 4·65 | 0·280 | 1·405 | 0·198 | — | — | — | 1·178 | 0·004 | 0·017 |
| 1·500 | 0·300 | 4·75 | 0·180 | 1·115 | 0·193 | — | — | — | 0·314 | — | 0·005 |

*F = fraction precipitated as hydroxide.

*Table 2. Relative abundance of the different species of chromium as a function of the sample pH obtained with the MECROM program.*

| pH | Cr(III) species (%) | | | | | Cr(VI species (%) | | | |
|---|---|---|---|---|---|---|---|---|---|
| | $Cr(H_2O)_6^{3+}$ | $Cr(OH)^{2+}$ | $Cr(OH)_2^+$ | $F$* | $Cr(OH)_4^-$ | $H_2CrO_4$ | $HCrO_4^-$ | $Cr_2O_7^{2-}$ | $CrO_4^{2-}$ |
| 3·00 | 89·88 | 10·08 | 0·03 | — | — | 0·54 | 99·36 | 0·12 | 0·03 |
| 4·00 | 46·35 | 52·01 | 1.64 | — | — | 0·05 | 99·56 | 0·13 | 0·32 |
| 4·50 | 20·39 | 72·37 | 7·23 | — | — | 0·01 | 98·90 | 0·13 | 1·01 |
| 5·00 | 6·34 | 71·16 | 22·50 | — | — | — | 96·80 | 0·13 | 3·07 |
| 5·50 | 1·39 | 49·30 | 49·31 | — | — | — | 90·67 | 0·11 | 9·27 |
| 6·00 | 0·02 | 2·48 | 7·85 | 89·63 | 0·01 | — | 75·52 | 0·07 | 24·42 |
| 6·50 | — | 0·03 | 0·29 | 99·63 | 0·04 | — | 49·40 | 0·03 | 50·57 |
| 7·00 | — | — | 0·01 | 99·88 | 0·11 | — | 23·59 | 0·01 | 76·39 |
| 8·00 | — | — | — | 98·96 | 1·04 | — | 3·00 | — | 97·00 |
| 9·00 | — | — | — | 89·64 | 10·36 | — | 0·31 | — | 99·69 |
| 10·00 | — | — | — | 67·27 | 32·73 | — | 0·03 | — | 99·97 |

*$F$ = Fraction of Cr(III) precipitated as hydroxide.

concentrations. Owing to the moderately acidic pHs of these samples the $H_2CrO_4$, $Cr(OH)_3$ and $Cr(OH)_4^-$ contents are almost nil. The predominant species at such pHs are $Cr(OH)^{2+}$ and $HCrO_4^-$ for Cr(III) and Cr(VI), respectively.

In a second series of experiments the influence of the pH on the distribution of these species was studied. With this purpose, synthetic samples were prepared with a constant concentration of both oxidation states of chromium and different pHs. Table 2 shows the relative abundance of the different Cr(III) and Cr(VI) species for pH values between 3 and 10.

### Discussion

This report demonstrates that it is possible to automate studies with a non-segmented flow system and a microprocessor. The system described provides a concentration profile of the different chemical forms in which an element can occur in natural or artificial samples. The most important advantages of this new technique for speciation studies are: rapidity, easy operation and economy. The suggested system can be applied to the speciation of such elements as iron, selenium, arsenic, etc. by using an appropriate chemical reaction.

Similarly to other papers on speciation based on numerical calculations [13 and 14], the results are approximate and give an idea of the distribution of the different chemical forms of the element under study. The results obtained in this paper indicate that for Cr(III) species $Cr(H_2O)_6^{3+}$ and $Cr(OH)_2^+$ co-exist at moderately acidic pH (the monohydroxylated species being the prevailing one) while at neutral or basic pHs, in which the insoluble hydroxide is formed, the species predominating in solution is $Cr(OH)_4^-$. For Cr(VI) the prevailing species are $HCrO_4^-$ in acidic medium and $CrO_4^{2-}$ in neutral or basic medium. Owing to the relatively small Cr(VI) concentration, the species $Cr_2O_7^{2-}$ does not surpass 0·15% of abundance in any sample.

### References

1. Burrows, D., (Ed) *Chromium Metabolism and Toxicity* (CRC Press, Inc., Florida, 1983).
2. Andrade, J. C., Rocha, J. C. and Baccan, N., *The Analyst*, **109** (1984), 645.
3. Jørgensen, S. S. and Regitano, M. A. B., *The Analyst*, **105** (1980), 292.
4. Bubnis, B. P., Straka, M. R. and Pacey, G. E., *Talanta*, **30** (1983), 30.
5. Lynch, T. P., Kernoghan, N. J., and Wilson, J. N., *The Analyst*, **109** (1984), 839.
6. Florence, T. M. and Batley, G. E. *CRC Critical Reviews in Analytical Chemistry*, **2** (1980), 219.
7 Andrade, J. C., Rocha, J. C. and Baccan, N., *The Analyst*, **110** (1985), 197.
8 Ruz, J. Ríos, A., Luque de Castro, M. D. and Valcárcel, M., *Analytica Chimica Acta* (in press).
9. Ibid, Fresenius, Z. *Analytische Chemie*, **322** (1985), 499.
10. Ibid, *Talanta* (in press).
11. Burriel, F., Lucena, F., Arribas, S. and Hernández, J., *Química Analítica Cualitativa* (Ed. Paraninfo, Madrid, 1982), pp. 595–598.
12. Tandon, P. T., Crisp, P. T., Ellis, J. and Baker, R. S., *Talanta*, **31** (1984), 227.
13. Stumm, W. and Morgan, J. J., *Aquatic Chemistry* (Wiley-Interscience, New York, 1970).
14. Jenne, E. A., *Chemical Modeling in Aqueous Systems* (American Chemical Society, Washington, D.C., 1979).

### Acknowledgement

The authors wish to acknowledge support for this research from Ministerio de Educación y Ciencia, through a grant from CAICyT No. 2012–83.

### Appendix

*Definition of variables (MECROM program)*

A = number of standards
AØ(I) = intercept of the calibration curve I
$A_1$(I) = slope of the calibration curve I
C(I) = concentration of standard I
$C_1$ = overall Cr(VI) concentration

$C_2$ = overall Cr(III) concentration

$C_3$ = concentration of $H_2CrO_4$

$C_4$ = concentration of $HCrO_4^-$

$C_5$ = concentration of $Cr_2O_7^{2-}$

$C_6$ = concentration of $CrO_4^{2-}$

$G_1$ = concentration of $Cr(H_2O)_6^{3+}$

$G_2$ = concentration of $Cr(OH)^{2+}$

$G_3$ = concentration of $Cr(OH)_2^+$

$G_4$ = fraction of Cr(III) precipited as hydroxide

$G_5$ = concentration of $Cr(OH)_4^-$

$K_1 \ldots K_7$ = equilibrium constants indicated in text

$L_1$ = counter of the calibration curves

M$ = 'S' when no more samples need to be analyzed

N = number of measurements

P = pH value

S = interval between measurements (ms).

### COMPUTING IN THE ANALYTICAL LABORATORY – WHERE HAVE WE GOT TO, WHERE ARE WE GOING?

A Joint Meeting of the East Anglia Region and the Automatic Methods Group of the Analytical Division of the Royal Society of Chemistry will be held on Tuesday, 29 April 1986, at New Hall, Cambridge, UK.

Papers include:

'Setting the scene – 21 years of laboratory computing', by A. M. C. Davies (AFRC Food Research Institute, Norwich, UK).

'Data capture and instrument control', by P. R. Fielden (UMIST, Manchester, UK).

'Data analysis', by B. Vandeginste (Katholieke University, Nijmegen, The Netherlands).

'Information systems', by A. Braithwaite (Trent Polytechnic, Nottingham, UK).

'Expert systems', by D. E. Wolstenholme (Imperial College, London).

'Robotic Systems', by D. Porter (Laboratory of the Government Chemist, London).

*More information from the Analytical Division, Royal Society of Chemistry, Burlington House, London W1V 0BN.*

### NOTES FOR AUTHORS

*Journal of Automatic Chemistry incorporating Journal of Clinical Laboratory Automation* covers all aspects of automation and mechanization in analytical, clinical and industrial environments. The *Journal* publishes original research papers; short communications on innovations, techniques and instrumentation, or current research in progress; reports on recent commercial developments; and meeting reports, book reviews and information on forthcoming events. All research papers are refereed.

### Manuscripts

Two copies of articles should be submitted. All articles should be typed in double spacing with ample margins, on one side of the paper only. The following items should be sent: (1) a title-page including a brief and informative title, avoiding the word 'new' and its synonyms; a full list of authors with their affiliations and full addresses; (2) an abstract of about 250 words; (3) the main text; (4) appendices (if any); (5) references; (6) tables, each table on a separate sheet and accompanied by a caption; (7) illustrations (diagrams, drawings and photographs) numbered in a single sequence from 1 upwards and with the author's name on the back of every illustration; captions to illustrations should be typed on a separate sheet. Papers are accepted for publication on condition that they have been submitted only to this *Journal*.

### References

References should be indicated in the text by numbers following the author's name, i.e. Skeggs [6]. In the reference section they should be arranged thus:

to a journal

Manks, D. P., *Journal of Automatic Chemistry*, **3** (1981), 119.

to a book

Malmstadt, H. V., in *Topics in Automatic Chemistry*, Ed. Stockwell, P. B. and Foreman, J. K. (Horwood, Chichester, 1978), p. 68.

### Illustrations

Original copies of diagrams and drawings should be supplied, and should be drawn to be suitable for reduction to the page or column width of the *Journal*, i.e. to 85 mm or 179 mm, with special attention to lettering size. Photographs may be sent as glossy prints or as negatives.

### Proofs and offprints

The principal or corresponding author will be sent proofs for checking and will receive 50 offprints free of charge. Additional offprints may be ordered on a form which accompanies the proofs.

*Manuscripts should be sent to either Dr P. B. Stockwell or Ms M. R. Stewart, see inside front cover.*

